# SensiPath: computer-aided design of sensing-enabling metabolic pathways

**DOI:** 10.1093/nar/gkw305

**Published:** 2016-04-22

**Authors:** Baudoin Delépine, Vincent Libis, Pablo Carbonell, Jean-Loup Faulon

**Affiliations:** 1 iSSB, Genopole, CNRS, UEVE, Université Paris Saclay, 91000 Évry, France; 2 Micalis Institute, INRA, AgroParisTech, Université Paris Saclay, 78350 Jouy-en-Josas, France; 3 SYNBIOCHEM Centre, Manchester Institute of Biotechnology, University of Manchester, M1 7DN Manchester, UK

## Abstract

Genetically-encoded biosensors offer a wide range of opportunities to develop advanced synthetic biology applications. Circuits with the ability of detecting and quantifying intracellular amounts of a compound of interest are central to whole-cell biosensors design for medical and environmental applications, and they also constitute essential parts for the selection and regulation of high-producer strains in metabolic engineering. However, the number of compounds that can be detected through natural mechanisms, like allosteric transcription factors, is limited; expanding the set of detectable compounds is therefore highly desirable. Here, we present the SensiPath web server, accessible at http://sensipath.micalis.fr. SensiPath implements a strategy to enlarge the set of detectable compounds by screening for multi-step enzymatic transformations converting non-detectable compounds into detectable ones. The SensiPath approach is based on the encoding of reactions through signature descriptors to explore sensing-enabling metabolic pathways, which are putative biochemical transformations of the target compound leading to known effectors of transcription factors. In that way, SensiPath enlarges the design space by broadening the potential use of biosensors in synthetic biology applications.

## INTRODUCTION

Synthetic biology and metabolic engineering applications often require as part of their design a way to assess the presence or to quantify the amount of a compound of interest. Genetically-encoded biosensors such as riboswitches and allosteric transcription factors offer the possibility to control the expression of a gene of choice. This feature makes them valuable for many applications (1,2) such as pollutant monitoring or high-throughput screening of optimized strains and enzymes (3–5), as expression of reporter genes like fluorescent proteins can be linked to the concentration of the compound of interest. Moreover, the ability of these biosensors to provide input at the genetic level opens the way to more complex downstream signal processing and actuation (6). Examples of applications of such circuits range from threshold activation in presence of pathological concentration levels of biomarkers (7) to the creation of a feedback control motif leading to yield improvement for a chemical producing strain (8).

There is thus a critical need for biosensors, but it appears that current strategies for finding new biosensors may not be sufficient to answer all the needs. Although remarkable progress has been made in the field of genetically encoded biosensor design (9–11) and genome mining (12), the number of chemicals that can be detected is still limited and thus constitute a bottleneck in the development of synthetic biology applications.

New strategies of biosensing can be considered to tackle this issue. One of them relies on indirect sensing by transforming the molecule of interest into a detectable one. Such strategy has been successfully used with the help of enzymes to transform a key metabolite such as L-tyrosine (13) or L-DOPA (14) into pigments and thus allowing high-throughput screening of overproducers. The same strategy can also be employed to transform the molecule of interest into a molecule for which a genetically-encoded biosensor is available (15,16). We recently demonstrated that this approach could be attempted in a systematic fashion by combining information on the available biosensors and automatic design of enzymatic networks. This led to the development of five new whole-cell biosensors for pollutants (parathion, 2C4NP), biomarker (hippuric acid) and drugs (cocain, nitroglycerin) (17).

In order to open this untapped source of biosensors for synthetic biologists, we hereby present SensiPath (http://sensipath.micalis.fr), a web-based tool assisting the design of sensing-enabling metabolic pathways (SEMPs). SensiPath will serve users wishing to perform cell-mediated detection of a compound when no direct-sensing solution is feasible. The primary objective of SensiPath, thus, is to enlarge the number of detectable compounds for synthetic biology applications. The algorithms we implemented to simulate biochemical reactions are derived from the well-tested RetroPath (18). It notably allows to take advantage of enzymatic promiscuity, i.e. the ability that enzymes have to process structurally similar substrates, thus yielding more results. SensiPath is built from a comprehensive list of more than 100 000 compounds and 87 000 reactions from four metabolic databases, covering most of the known metabolism. We also collected a large dataset of more than 500 detectable compounds for which intracellular biosensors exist from several gene expression regulation databases, focusing our search on allosteric transcription factors.

## MATERIALS AND METHODS

**Figure 1.**
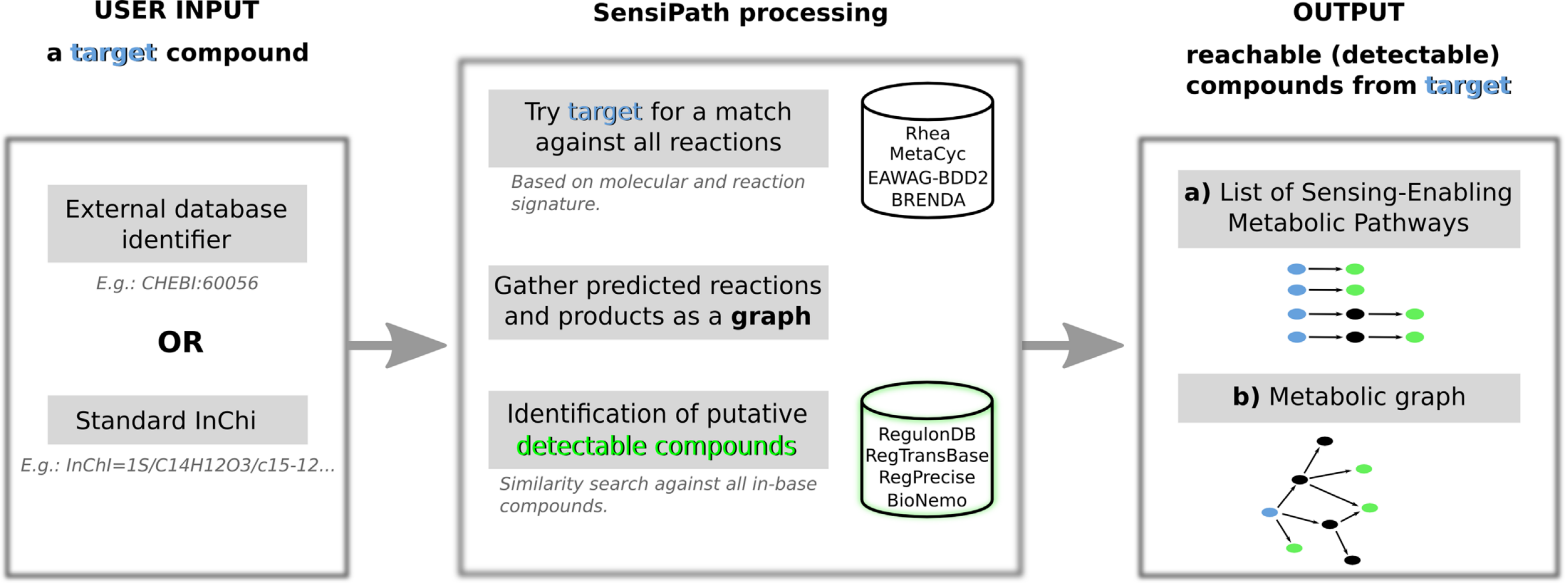
SensiPath flowchart. Users query a target compound (blue), either as an external database identifier or as a standard InChi. Target is processed to gather reachable compounds through enzymatic reactions; detectable compounds are annotated (green). Results are displayed both as (a) the set of pathways leading to recognized detectable compounds; and (b) the whole computed graph around the target.

Figure 1 shows an overview of how SensiPath works, the details are exposed in the following subsections. SensiPath is based on a comprehensive internal database of biochemical reactions and compounds encoded as chemical signatures. Once a compound query is submitted, it performs a search in order to find a match against all the enzymatic reactions that we have collected in our database. The search is carried out in order to predict reachable compounds from the target. This search generates a metabolic graph at up to two enzymatic steps away from the target, in which nodes are compounds and edges are reactions. Detectable compounds are identified and annotated by a score of similarity based on searching against the list of known detectable compounds in the database. For later reference, all SensiPath sources in its current online version are available on FigShare (https://dx.doi.org/10.6084/m9.figshare.3144616.v1) in addition of our list of detectable compounds (https://dx.doi.org/10.6084/m9.figshare.3144715.v1).

### Source databases

SensiPath predictions are based on imported data from metabolic and gene expression regulation databases. We gathered data from multiple sources to cover most of available knowledge in current databases.

#### Reactions

Known biochemical reactions were extracted from main common reaction databases (Rhea (v66, http://www.rhea-db.org) (19), MetaCyc (v19.1, http://metacyc.org) (20), BRENDA (v15.2, http://www.brenda-enzymes.info) (21) as well as from a more specialized database, the Biocatalysis/Biodegradation Database (http://eawag-bbd.ethz.ch, accessed in December 2015) (22). We considered only reactions for which structures of all reactants were available, fully defined and valid. Overall, we collected more than 100 000 compounds and 87 000 reactions with references to external databases.

#### Detectable compounds

We gathered a list of 504 putative detectable compounds focusing our search on effectors of allosteric transcription factors from prokaryotes. Data were collected from several gene expression regulation databases: RegulonDB (v9.0, http://regulondb.ccg.unam.mx) (23), RegPrecise (v4, http://regprecise.lbl.gov) (24), RegTransBase (v7, http://regtransbase.lbl.gov) (25) and BioNemo (v6.0, http://bionemo.bioinfo.cnio.es) (26).

### Reaction and compound encoding

In order to encode the reactions we first normalized the compounds, next computed molecular signatures and finally computed reaction signatures.

#### Compound normalization

The representation of compounds must be normalized in order to improve the performance of the encoding method. In particular, compounds were represented under their aromatic form while charges and hydrogens were removed; stereochemistry was kept.

#### Molecular signature

All compounds were encoded internally through their molecular signature (27). The molecular signature of a compound is a list of overlapping molecular fragments, each of them centred on a distinct atom. Thus, fragments represent atom neighbourhood (also called atomic signature or atomic environment) in terms of atom and bond type. Basically, a molecular signature is similar to the extended connectivity circular fingerprint (ECFP) (28). We used fragments (atomic signatures) with an environment diameter of 12 bonds.

#### Reaction signature

All biochemical reactions were represented internally by reaction signatures (29). The reaction signature σ(R) is defined in a vector space as the sum of molecular signatures of products less the sum of molecular signatures of substrates:

\begin{equation*}{}^d\sigma ({R_n}) = \sum\limits_i {{}^d\sigma ({P_i})} - \sum\limits_j {{}^d\sigma ({S_j})} \end{equation*}

where ^*d*^*σ*(*P_i_*) and ^*d*^*σ*(*S_j_*) are the molecular signatures of substrate *S_j_* and product *P_i_* at diameter *d*.

This approach allows us to encode biochemical reactions by looking at the changes occurring at the reaction center. Note that the specificity of a reaction signature is determined by the diameter of the molecular signature, as lower diameters encode multiple compounds while higher diameters are specific. Therefore, reaction signatures have been shown as a handy way to model enzymatic substrate promiscuity (18,29,30), i.e. the ability that enzymes have to process structurally similar substrates. Our chosen diameter of 12 assumes a relatively low degree of enzymatic promiscuity for the encoded reactions.

### Matching algorithm

After integrating reaction signatures in our database, we can predict on-the-fly if a compound can act as substrate of a reaction by using a new implementation of the RetroPath forward algorithm (18). If a compound C has a list of fragments (atomic signatures and their respective occurrence) embedding the substrate fragments contained in a reaction signature R (i.e. the negative part of reaction signature), then the compound is said to match the reaction. The sum of the signatures of compound C and those of the reaction R generates a new list of (positive) fragments P, representing the putative products generated by the reaction signature acting upon compound C. If we can retrieve a set of known compounds from those fragments, then the reaction is accepted and C is considered a valid substrate for R to produce P.

### Metabolic graph

Pathways are handled as a graph (where nodes are compounds and edges reactions) with NetworkX python library (31).

### Similarity search

In order to annotate compounds structurally similar to detectable compounds in predicted metabolic graphs, we precomputed the similarities between all compounds and detectable ones. Indeed, promiscuous detection of structurally similar compounds may not be reported in databases and should be checked in the literature if no suitable detectable compound is found by SensiPath.

Similarity was evaluated with RDKit python library (http://www.rdkit.org/), representing compounds with RDKit's ECFP4 fingerprint implementation and a Jaccard-Tanimoto index (32). A Tanimoto of one is a perfect match.

### Web server implementation

SensiPath web server is a Docker application running the following standard software packages: Nginx, gUnicorn, Django and Postgres. Data and matching functions are stored in the database.

## INPUT AND OUTPUT

### Input

Users query SensiPath with the compound they wish to detect (Figure 1, left panel), either as an identifier from an external database (e.g. ChEBI available at https://www.ebi.ac.uk/chebi/) or as a standard InChi (http://www.inchi-trust.org/). InChi is a IUPAC string representation of compounds and can be easily obtained from compound databases. Users can choose to search for detectable compounds that are at one or two enzymatic steps away from their target.

### Output

**Figure 2.**
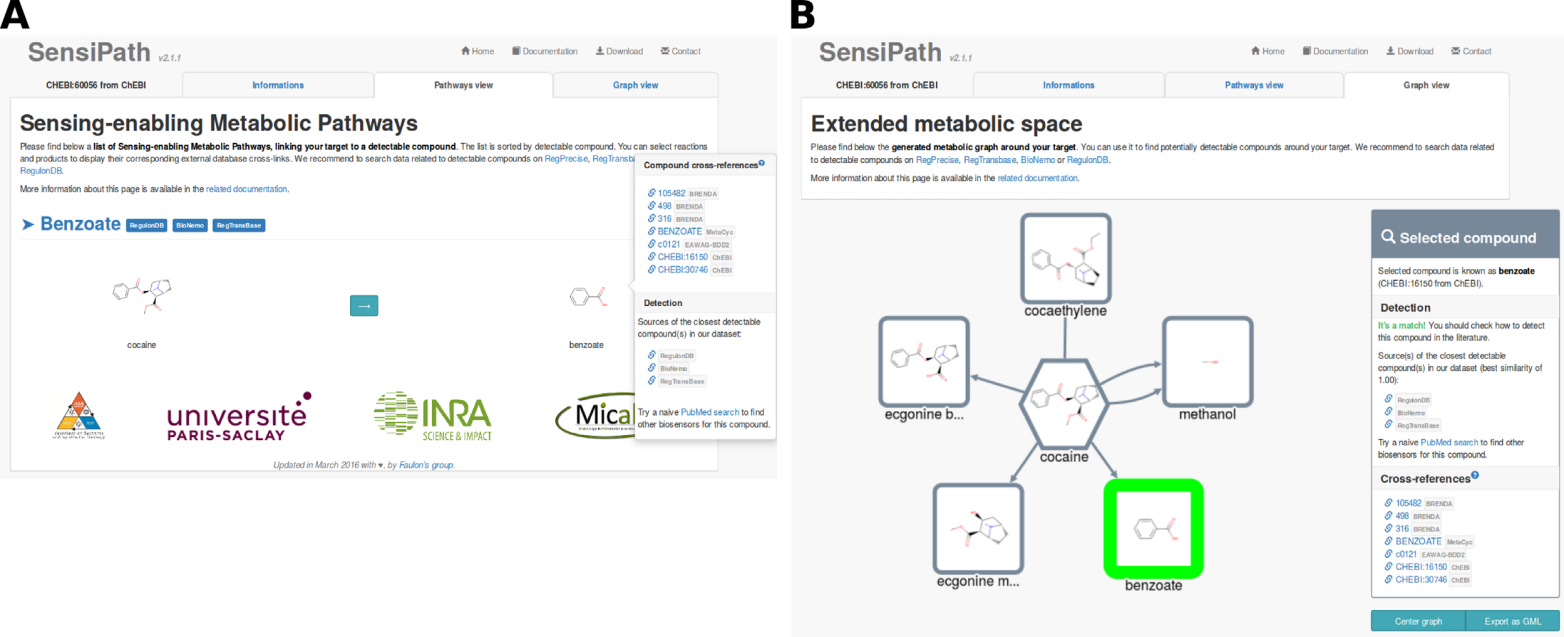
Results pages for querying ‘CHEBI:60056’ at one step. (**A**) Pathway view. List of sensing-enabling metabolic pathways, sorted by detectable compound. Clicking reactions (arrows) or products will display a list of cross-references to relevant databases. Cross-references which are displayed together are considered identical by SensiPath. (**B**) Graph view. Interactive computed graph around the target (hexagonal node). Putative detectable compounds have a green border. Selected elements (edge or node) have a bold border. Again, clicking elements will display their respective cross-references (right panel). Note that reactions leading to several products have duplicated edges; for instance, benzoate and ecgonine methyl ester are both products of the same biochemical reaction (hydrolysis by a cocaine esterase).

SensiPath displays its results in two views; (i) pathway view: the set of pathways leading to recognized detectable compounds (Figure 2A); and (ii) graph view: the whole computed graph around the target (Figure 2B), also available for download as a standard Graph Markup Language file.

## CASE STUDIES

Five examples of SEMPs were characterized experimentally in *Escherichia coli* by our group to validate SEMP concept. For a model bacteria such as *E. coli, in vivo* implementation of SEMPs only requires basic molecular biology knowledge. As an example, we describe here the design steps required to build a strain of *E. coli* able to detect the drug cocaine, and a strain able to detect the pollutant parathion with the help of SensiPath. A detailed *in vivo* characterization for these examples is described elsewhere (17).

We refer to ‘the metabolic module’ as the genetic parts providing the enzymatic transformations and to ‘the sensing module’ as the gene circuit consisting of the transcription factor, its responsive promoter and the reporter gene.

### Cocaine detection

While several studies have shown interest in detecting cocaine in biological samples, they rely on aptamers and nanoparticles sensors (33,34), which do not allow the signal to be transferred to the genetic layer of a living organism, a requirement for further *in situ* signal processing.

Here, we show how SensiPath was used in order to design a SEMP that detects cocaine *in vivo*. To that end, SensiPath web server is queried using a chemical identifier of cocaine, either through CHEBI:60056 or InChi=1S/C17H21NO4/c1-18-12-8-9-13(18)15(17(20)21-2)14(10-12)22-16(19)11-6-4-3-5-7-11/h3-7,12-15H,8-10H2,1-2H3/t12-,13+,14-,15+/m0/s1. SensiPath founds a candidate SEMP allowing detection that is one enzymatic step away from the target. On the Graph view (Figure 2B), the five different products obtained through known enzymatic activities on cocaine are displayed. Clicking on an edge of the graph provides a link to databases providing information on each reaction. One of these compounds has a green border indicating that a biosensor is known to interact with an identical or highly similar chemical structure. This suggests that the information of the presence of cocaine in the medium can be transferred to the genetic layer and thus constitutes a putative SEMP. All found SEMPs are summarized on the Pathway view (Figure 2A). In the present case study, cocaine can be hydrolysed and forms the detectable molecule benzoate. Clicking on the arrow that represents the enzymatic transformation will display cross reference links to external databases of enzymatic transformations. It is strongly recommended to carefully check the bibliography that motivated the annotation of the reaction in the database, since important results might be omitted or misrepresented due to an incorrect curation process. In the case of cocaine hydrolysis, several publications confirm the benzoate conversion and databases such as Rhea and MetaCyc provide a direct link to Uniprot or GenBank where the sequence coding for the enzyme can be found (GenBank AF173165.1). This sequence can be synthesized and cloned into an expression vector of choice to constitute the metabolic module part of the SEMP.

In parallel, a query on BioNemo or RegTransBase for benzoate (or benzoic acid), the compound reported by SensiPath as having a biosensor, leads to several potential transcription factors that are known to interact with this compound (BenM, BenR, CbdS, PcaR, TcbR, CatR, BadR and XylS). In our experimental implementation, we chose the couple composed of BenR and its responsive promoter pBen from *Pseudomonas putida* KT2440 after a quick assessment of the available literature.

The sequence of pBen can then be synthesized and cloned in front of a reporter gene of choice (e.g. a fluorescent protein) in addition to the transcription factor coding sequence in order to form the sensing module part of the SEMP. To maximize the chances of proper expression of the heterologous proteins, we recommend to perform a step of codon-optimization on all the coding sequences, to place them under control of inducible promoters and to use strain such as BL21(DE3) due to its efficient protein expression capabilities.

### Parathion detection

Synthetic biology application of biosensors in the field of environmental protection could take the form of micro-organisms programmed with a ‘seek and destroy’ behaviour toward pollutants (35). However, the task of engineering tailor-made biosensors for pollutants has been difficult to date (36).

Parathion is listed as one of the twelve worst offenders persistent organic pollutants according to the United Nations Environment Program and could benefit from such synthetic biology applications provided that a biosensor is available.

A request on Sensipath for parathion, with identifier CHEBI:27928, leads to the identification of a 1-step SEMP that depends on a phosphotriesterase (PTE) allowing transformation of parathion into 4-nitrophenol. As in the previous cocaine example, the proposed transformation could be verified in the literature (37). We have experimentally validated this SEMP with a metabolic module based on the PTE coding sequence coupled with the sensing module made up of the transcription factor DmpR and its responsive promoter Pu from Pseudomonas sp. CF600. However, both PTE enzyme and DmpR promoter are known to be promiscuous, and other pollutants harbouring phenolic structures could activate DmpR. As this could impair applications requiring a high specificity, alternative SEMPs were also explored.

Interestingly, with a 2-steps query, SensiPath's Pathway view shows that 4-nitrophenol can be an intermediate compound to another SEMP based on nitrite detection. Indeed a second enzymatic step mediated by a monooxygenase (38) is able to further transform 4-nitrophenol into nitrite, which is known to interact with regulators such as NarL from *E. coli*. This alternative offers the possibility of developing a more specific biosensor, effectively discarding any risk of cross-activation by phenolic compounds, as long as they do not have a nitro group. Going further with this idea, high specificity target detection could be guaranteed by building up combinations of alternative SEMPs in one or several strains.

## DISCUSSION

The development of novel biosensors is presently needed in order to enlarge the set of detectable and observable metabolites that are available for synthetic biology applications such as in health, environment or fine chemical production. In that direction, the SensiPath web server provides synthetic biologists with new solutions to build circuits having the ability of triggering a genetic response when a compound of interest is present. Our biosensor design solution is based on the strategy, not fully explored previously, of performing an *in silico* screening for enzymatic pathways linking the target to known detectable compounds. The originality of the approach lies in the systematic search through a full enumeration that SensiPath carries out, allowing discovery of novel sensing pathway candidates in the metabolic space. Resulting SEMPs are appealing for synthetic biologists because they can be easily built using conventional DNA assembling techniques and tested *in vivo*. SensiPath thus provides an easy way to explore right out of the box multiple biosensor constructs.

Depending on the application, the reliability of the candidate SEMPs identified by our method may vary. Limitations of the SEMP method include the need for the target compound to be able to co-localize with the enzyme (i.e. to enter the cells or to be internally produced in the cell), and the need for enzymatic products of the sensing pathway to be not too toxic to the cell. Such issues need to be addressed in a case-by-case manner, since they greatly depend on the application and on physico-chemical properties that are not always known for the compound. Other potential limitations of the method hold with regards to the choice of the biosensor. Although some information about the degree of promiscuity of transcription factors may be available from databases and literature, this aspect should be carefully considered in each application, especially if the final application requires a high level of specificity. The choice of the biosensor should also take into account dose response parameters such as the dynamic range and linear range of detection. SEMP's properties will depend on the actual properties of the biosensor, an information that therefore should be considered and retrieved from the available literature. In addition, promoter sequences responding to transcription factors may not be always found in databases, often requiring an investigation of associated references. This information nevertheless is progressively becoming more available through repositories like the Registry of Standard Biological Parts (http://parts.igem.org/Main_Page).

In conclusion, we believe that the SEMP detection method is an interesting alternative worth considering with respect to tailored solutions such as rational design (10,11) or genome mining (12). To the authors acknowledgement, this is the first time a web-based tool is proposed to design biosensors based on the SEMPs approach. Other tools (such as M-path (39) or BioSynther (40) to name a few) proposed finding pathways from one compound to another, but they did not include any detectability concept in the way it was considered here. In that sense, SensiPath and SEMPs will surely contribute to the design of new synthetic biology applications. Moreover, we should expect in the next years to see the broadness of applicability of SEMPs to increase in parallel with progress in reaction and gene expression regulation knowledge sources.

## AVAILABILITY

SensiPath is available online at http://sensipath.micalis.fr. A stand-alone snapshot of SensiPath at the time this manuscript was written is available on FigShare at https://dx.doi.org/10.6084/m9.figshare.3144616.v1.
